# Altered Relationship Between Heart Rate Variability and fMRI-Based Functional Connectivity in People With Epilepsy

**DOI:** 10.3389/fneur.2021.671890

**Published:** 2021-06-10

**Authors:** Michalis Kassinopoulos, Ronald M. Harper, Maxime Guye, Louis Lemieux, Beate Diehl

**Affiliations:** ^1^Department of Clinical and Experimental Epilepsy, Institute of Neurology, University College London, London, United Kingdom; ^2^Epilepsy Society, Buckinghamshire, United Kingdom; ^3^Brain Research Institute, University of California, Los Angeles, Los Angeles, CA, United States; ^4^Department of Neurobiology, David Geffen School of Medicine, University of California, Los Angeles, Los Angeles, CA, United States; ^5^Aix Marseille Univ, CNRS, CRMBM, Marseille, France; ^6^APHM, Hôpital Universitaire Timone, CEMEREM, Marseille, France

**Keywords:** state-dependent functional connectivity, sympathovagal balance, SUDEP, thalamic connectivity, ventral attention network, insula cortex

## Abstract

**Background:** Disruptions in central autonomic processes in people with epilepsy have been studied through evaluation of heart rate variability (HRV). Decreased HRV appears in epilepsy compared to healthy controls, suggesting a shift in autonomic balance toward sympathetic dominance; recent studies have associated HRV changes with seizure severity and outcome of interventions. However, the processes underlying these autonomic changes remain unclear. We examined the nature of these changes by assessing alterations in whole-brain functional connectivity, and relating those alterations to HRV.

**Methods:** We examined regional brain activity and functional organization in 28 drug-resistant epilepsy patients and 16 healthy controls using resting-state functional magnetic resonance imaging (fMRI). We employed an HRV state-dependent functional connectivity (FC) framework with low and high HRV states derived from the following four cardiac-related variables: 1. RR interval, 2. root mean square of successive differences (RMSSD), 4. low-frequency HRV (0.04–0.15 Hz; LF-HRV) and high-frequency HRV (0.15–0.40 Hz; HF-HRV). The effect of group (epilepsy vs. controls), HRV state (low vs. high) and the interactions of group and state were assessed using a mixed analysis of variance (ANOVA). We assessed FC within and between 7 large-scale functional networks consisting of cortical regions and 4 subcortical networks, the amygdala, hippocampus, basal ganglia and thalamus networks.

**Results:** Consistent with previous studies, decreased RR interval (increased heart rate) and decreased HF-HRV appeared in people with epilepsy compared to healthy controls. For both groups, fluctuations in heart rate were positively correlated with BOLD activity in bilateral thalamus and regions of the cerebellum, and negatively correlated with BOLD activity in the insula, putamen, superior temporal gyrus and inferior frontal gyrus. Connectivity strength in patients between right thalamus and ventral attention network (mainly insula) increased in the high LF-HRV state compared to low LF-HRV; the opposite trend appeared in healthy controls. A similar pattern emerged for connectivity between the thalamus and basal ganglia.

**Conclusion:** The findings suggest that resting connectivity patterns between the thalamus and other structures underlying HRV expression are modified in people with drug-resistant epilepsy compared to healthy controls.

## Introduction

Heart rate varies on a moment-to-moment basis in response to changing physiological demands, and is regulated by sympathetic and parasympathetic components of the autonomic nervous system (ANS). Evaluation of the momentary changes in heart rate variability (HRV) can provide insights into the interplay of central mechanisms controlling sympathetic and parasympathetic (vagal) activity (1, 2). A shift toward parasympathetic dominance is typically accompanied by heart rate declines and increased HRV; whereas, increased sympathetic dominance is typically associated with an accelerated heart rate and decreased HRV [although deviations from this generality occur (3)]. Considerable evidence exists that HRV provides an indication of sympathovagal balance and can be useful as a marker for certain cardiovascular diseases (4), mortality, and sudden death (5).

Epilepsy is accompanied by significantly different patterns of HRV (6). A lower interictal HRV is often reported in drug-resistant epilepsy, suggesting a shift toward sympathetic predominance (6–9). In addition, a link between peri-ictal HRV and major motor seizure severity (10) has been outlined, as well as an indication of seizure reduction following vagal nerve stimulation in patients with drug-resistant epilepsy (11). HRV determination of low parasympathetic activity and increased risk of sudden unexpected death in epilepsy SUDEP has been described (12, 13), as well as altered circadian rhythms of HRV in epilepsy (14, 15); the latter finding may explain the larger number of night-time SUDEP cases (16). However, a poor understanding of the mechanisms underlying expression of cardiac functions in epilepsy hampers interpretation of alterations in brain regulatory sites controlling HRV and the potential to gain insights into dysfunctions within those processes.

Functional magnetic resonance imaging (fMRI), a non-invasive tool for probing brain activity and functional connectivity (FC), has been used to study the neural substrates of autonomic regulation (17–20). Initial studies primarily relied on tasks to excite the ANS (21–26), while subsequent studies have used resting-state fMRI (27–29), which has a benefit of not being confounded by task-related changes in local brain activity and FC. Differences in HRV across participants as well as fluctuations in HRV within-individuals have been related to spontaneous regional blood-oxygen-level-dependent (BOLD) fluctuations and connectivity between distinct regions (27–29). Regions found in fMRI studies to be associated with autonomic regulation, such as the anterior cingulate (ACC), medial prefrontal (mPFC) and insular cortices, form part of the central autonomic network (CAN) described in preclinical studies, a system of brain structures involved in ANS functions (30, 31).

Functional connectivity measures between brain sites are altered in people with epilepsy (32–36); however, it is unclear whether these alterations are linked to impaired cardiac regulation. Here, we investigated alterations in brain functional organization in relation to cardiac rhythms in people with epilepsy. We employed an HRV state-dependent FC framework with two levels of variability states estimated from electrocardiogram (ECG) recorded during resting-state fMRI. Given the association between HRV measures and time-varying FC reported in the literature (27), a state-dependent FC framework informed by concurrent cardiac recordings appeared more suitable for studying cardiac dysfunction than static FC approaches that do not utilize physiological recordings. Moreover, we examined whole-brain FC in a data-driven manner rather than restricting the analysis to interactions between regions of the CAN, as recent studies suggested that the neural correlates of cardiac regulation are more widespread than initially thought (17, 29, 37).

## Materials and Methods

### Subjects

Thirty-two (32) patients with drug-resistant epilepsy were selected from an ongoing investigation into the localization of epileptic activity in the brain using simultaneous EEG-fMRI with ECG (38), with a case ascertainment period between 2005 and 2014. The inclusion criteria were: (1) the availability of a resting-state EEG-fMRI scan; and (2) a high-resolution T1-weighted scan. The exclusion criteria were: (1) large brain lesion or previous neurosurgery [we considered large to be anything greater than a small area of focal cortical dysplasia (FCD) or sclerosis – e.g., tumors, cavernomas] (2) incomplete clinical or imaging data (e.g., abandoned scans). Sixteen (16) healthy controls were also considered with comparable age and sex characteristics; the group demographics and clinical details are shown in Supplementary Tables 1, 2. The study was approved by the National Research Ethics Committee (United Kingdom; 04/Q0502/89) and all patients gave written informed consent.

### Simultaneous EEG-fMRI Acquisition

Scanning was performed at the Epilepsy Society (Chalfont St Peter, Buckinghamshire, UK) on a 3.0 Tesla GE (Signa excite HDX) scanner. A 20-min (400 vol) ${\text{T}}_{2}^{*}$-weighted fMRI scan was collected from each subject except for two patients that were scanned for 10-min instead. The fMRI scan was done using a gradient-echo echo-planar-imaging with the following characteristics: repetition time (TR) = 3,000 ms, echo time (TE) = 30 ms, flip angle = 90, matrix size = 64 × 64, field of view (FOV) = 24 × 24 cm^2^, slice thickness = 2.4 mm with 0.6 mm gap, 44 slices, and voxel size = 3.75 × 3.75 × 3 mm^3^. Subjects were instructed to keep their eyes closed, avoid falling asleep, and not think about anything in particular. A T_1_-weighted image was also acquired using an FSPGR (fast spoiled gradient recalled echo) sequence, with the following parameters: matrix size = 256 × 256, FOV = 24 × 24 cm^2^, slice thickness = 1.5 mm, 150 slices, and voxel size = 0.94 × 0.94 × 1.5 mm^3^.

Scalp EEG signals and an ECG signal were simultaneously acquired during fMRI scanning at 5 kHz using a 64 channel MR-compatible EEG system with ring Ag/AgCl electrodes (BrainAmp MR+; Brain Products GmbH, Munich, Germany). The electrodes were placed according to the 10/20 system and referenced to electrode FCz.

### Preprocessing of fMRI Data

As described previously (38), preprocessing of fMRI data was conducted using the Statistical Parametric Mapping software (SPM12, Welcome Trust Centre for Neuroimaging, London, UK, http://www.fil.ion.ucl.ac.uk/spm) (39) in a Matlab environment (R2020a; Mathworks, Natick, Massachusetts, USA). The first five functional volumes were discarded to allow steady-state magnetization to be established, and the remaining volumes were realigned to correct for head movements. The structural image of each subject was co-registered to the mean realigned functional volume and, subsequently, underwent tissue segmentation into gray matter, white matter and cerebrospinal fluid tissue compartments. The functional images as well as the coregistered structural images and tissue compartment masks were spatially normalized to the Montreal Neurological Institute (MNI) reference space using non-linear transformation.

To account for anatomical variability across participants and reduce thermal noise, all individual functional volumes were smoothed using a 5 mm full-width half-maximum (FWHM) Gaussian kernel. Subsequently, the Brainnetome atlas was used to extract mean fMRI time-series from 210 cortical and 36 subcortical parcels (40). The parcel time-series were high-pass filtered at 0.008 Hz to avoid spurious correlations that arise from low-frequency fluctuations (41).

We used the framewise displacement (FD) as defined in Power et al. (42) to identify and exclude subjects with high levels of motion, as motion can obscure neural-related BOLD activity (43, 44) and lead to systematic biases in FC studies (45–47). FD is calculated from the six motion realignment parameters and reflects the extent of motion at each timepoint. Subjects that were characterized by mean FD larger than 0.25 mm were excluded. In addition, for the remaining of the subjects that were considered in the study, timepoints with FD larger than 0.2 mm were disregarded.

Finally, to further mitigate the effects of motion as well as reduce the effects of physiological processes and scanner artifacts, we regressed out the following nuisance regressors from all parcel time-series: the first ten principal components from voxel time-series within the white matter (48), six regressors related to cardiac pulsatility artifacts obtained with the convolution model proposed in Kassinopoulos and Mitsis (45), and the mean fMRI time-series averaged across all voxels within the gray matter.

### Preprocessing of ECG and Calculation of HRV Measures

The ECG was corrected for gradient artifacts using adaptive template subtraction (49) implemented in BrainVision Analyzer 2 software (Brain Products GmbH, Munich, Germany), and band-pass filtered from 0.5 to 40 Hz. The R-waves were detected using Matlab's function findpeaks with a minimum peak distance varying between 0.5 and 0.9 s depending on the subject's average RR interval (time between successive R-waves).

The RR intervals were used to obtain time-series of the root mean square of successive differences in RR intervals (RMSSD), and the normalized low (0.04–0.15 Hz) and high (0.15–0.40 Hz) frequency components of HRV. The aforementioned three HRV measures were computed in adjacent time windows of 100 s each, and a timestep of 1 s, to probe changes in sympathetic and parasympathetic activity during the 20-min resting-state scan. RMSSD is a time-domain HRV measure that is believed to reflect parasympathetic activity (50), the low-frequency HRV (LF-HRV) is a frequency-domain measure presumably sensitive to both branches of the ANS, and the high-frequency HRV (HF-HRV) is a frequency-domain measure that, similar to RMSSD, reflects parasympathetic activity. To derive the normalized LF- and HF-HRV measures, the time-series of successive differences in RR intervals was uniformly resampled at 10 Hz before estimating the Welch power spectral density. Subsequently, the power within the frequency ranges 0.04–0.15 Hz (LF-HRV) and 0.15–0.40 Hz (HF-HRV) was divided by the power within the range 0.04–0.50 Hz and multiplied by 100%. Apart from the three HRV measures, the moving average of RR intervals was also computed across the time windows. Before the calculation of an HRV measure or mean RR interval within a time window, outliers of RR values, defined as three median absolute deviations (MAD) away from the median, were linearly interpolated. To disentangle fluctuations in HRV from fluctuations in RR interval, the three HRV measures were orthogonalized with respect to fluctuations in RR interval. Furthermore, heart rate was estimated as the inverse of instantaneous RR interval multiplied by 60. The heart rate was preferred over the instantaneous RR interval to facilitate comparison with the activation maps shown in Valenza et al. (29) that link regional BOLD fluctuations to heart rate.

### Relationship Between BOLD Fluctuations and Cardiac Dynamics

The three HRV measures (RMSSD, LF-HRV and HF-HRV), moving-average RR interval and instantaneous heart rate were convolved with the canonical hemodynamic response function (HRF) from SPM12 (39) prior to resampling at the fMRI acquisition rate. The association between the obtained time-series and voxel-wise fMRI time-series within the gray matter was quantified using one-sample *t*-test on the associated beta parameters derived from the general linear model. Differences in the activation maps between epilepsy patients and healthy controls were assessed with a second-level, mixed-effects analysis with subjects as the random-effects factor, using a two-sample *t*-test on the associated beta parameters. To control for potential effects, sex, age and levels of head motion (i.e., mean FD) were treated as covariates. Moreover, to account for false positives, the statistical maps were thresholded with a voxel-wise threshold of family-wise error (FWE) rate *p* < 0.05 corrected for multiple comparisons using the random-field theory (51) and an extent threshold ≥ 10 voxels.

### Whole-Brain HRV State-Dependent Functional Connectivity

To investigate the effects of HRV state on brain functional organization, a whole-brain state-dependent FC analysis was performed on the parcellation of the Brainnetome atlas that includes 246 parcels covering the neocortex and sub-cortical regions (40). The connectivity strength between parcel pairs was determined based on the pairwise Pearson correlation coefficient of the parcel time-series. Cortical parcels were grouped into the following seven large-scale functional networks described in Yeo et al. (52): visual (34 parcels), somatomotor (33 parcels), dorsal attention (30 parcels), ventral attention (22 parcels), limbic (26 parcels), frontoparietal (26 parcels) and default mode (36 parcels), using the mapping provided on the Brainnetome atlas' website (https://atlas.brainnetome.org/), and the subcortical parcels were grouped into the following four networks: amygdala (four parcels), hippocampus (four parcels), basal ganglia (12 parcels) and thalamus (16 parcels) (three cortical parcels were excluded from the analysis as they were not assigned to any of the networks). To better understand how FC depends on the state of autonomic activity, we estimated FC in each individual considering low or high HRV states separately. The low and high HRV states were defined as the timepoints in a scan at which an HRV measure (e.g., RMSSD) had values in the lowest and highest quartile range for that given scan, respectively.

A mixed analysis of variance (ANOVA) was conducted with the group (epilepsy patients / healthy controls) as a between-subject factor and HRV state (low/high) as a within-subject factor, which allowed us to examine the effect of the group and HRV state on FC within and between networks as well as their interactions. Potential effects of sex, age and levels of head motion on FC were regressed out through linear regression at the group level before conducting the mixed ANOVA. The levels of head motion for the low and high HRV state were determined separately considering only the timepoints corresponding to each state. The connectivity strength between pairs of networks that was used in the mixed ANOVA was defined as the mean correlation averaged across all pairs of parcels that belonged to the two corresponding networks. The HRV state-dependent FC analysis was performed for the three HRV measures and the moving-average RR interval. Statistical significance was set at *p* < 0.0125 (i.e., 0.05/4) adjusted for multiple comparison with respect to pairs of networks using false discovery rate (FDR).

## Results

Data from four patients were excluded due to excessive motion (mean FD > 0.25 mm). The sex and age distributions were similar between the epilepsy patients (*n* = 28, mean age of 28.7, 14 women) and healthy controls (*n* = 16, mean age of 30.6, seven women;) (*p* > 0.48; two-sample permutation test; number of permutations *q* = 10,000; Supplementary Table 2). Motion-contaminated fMRI volumes (FD > 0.2 mm) were also excluded, resulting in an average of 353 ± 65 volumes per subject. Based on a two-sample permutation test (*q* = 10,000) there were no significant differences in the number of volumes between healthy controls and epilepsy patients (*p* > 0.10), and the two groups exhibited similar levels of motion during the fMRI scan (*p* > 0.10; Supplementary Figure 1). The consideration of a subset of volumes for the low and high HRV states (average 88 volumes per state) did not have any apparent effects on the estimates of whole-brain FC as compared to the FC matrices obtained from the entire scan (Supplementary Figure 2).

### Heart Rate and HRV Measures

**Figure 1:**
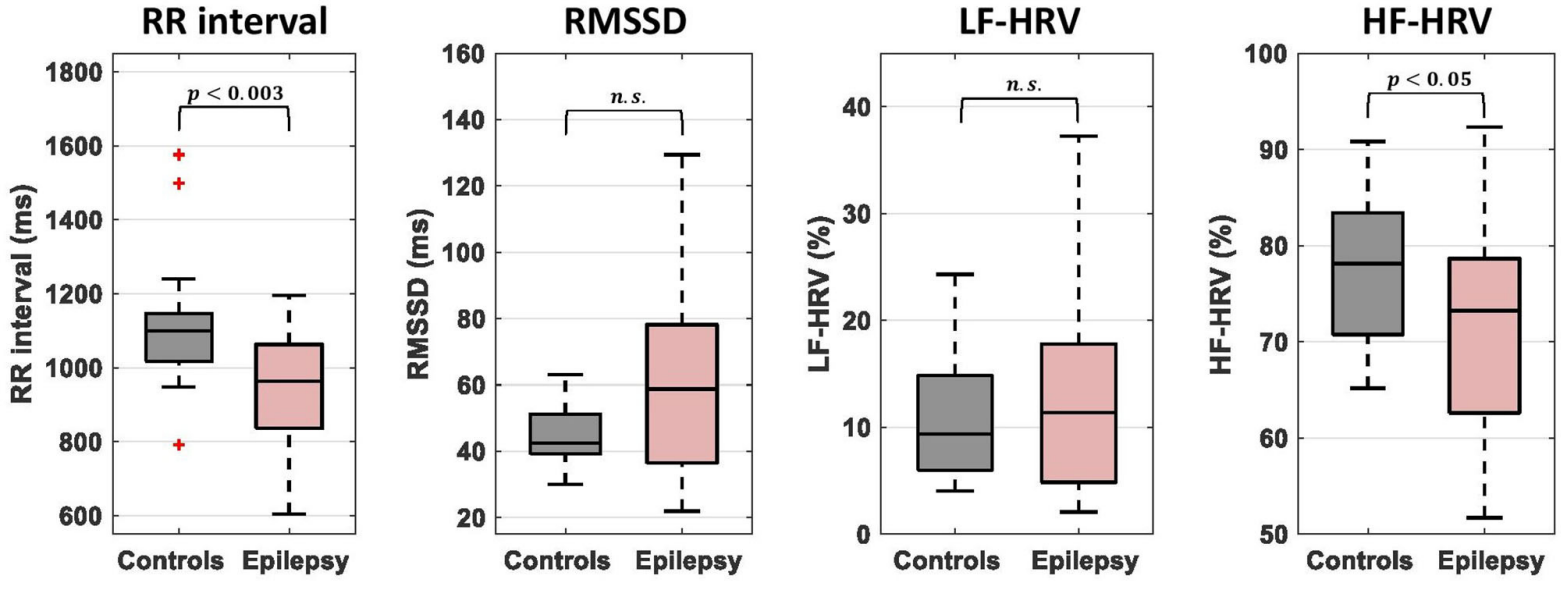
Comparison of mean RR interval and heart rate variability (HRV) measures between epilepsy patients and healthy controls. The bottom and top of each box correspond to the 25th and 75th percentiles of the sample distribution, the line in the box corresponds to the median and the crosses indicate outliers, defined as values that are more than 1.5 times the interquartile range away from the edges of the box. The epilepsy patients showed significantly lower values of mean RR interval and HR-HRV than healthy controls. n.s., not significant.

Comparisons of cardiac dynamic metrics between patients and healthy controls were performed using a two-sample permutation test (*q* = 10,000) after regressing out potential effects of sex and age. The mean RR interval during the 20-min scan was significantly lower in patients compared to healthy controls (950 ± 100 ms vs. 1,100 ± 200 ms; *p* < 0.003; equivalently, the mean heart rate was significantly higher) (Figure 1). HF-HRV was also lower in epilepsy compared to controls (71 ± 11% vs. 78 ± 8 %; *p* < 0.05) whereas RMSSD and LF-HRV were similar in the two groups. The LF-HRV (0.04–0.15 Hz) and HF-HRV (0.15–0.40 Hz) measures demonstrated a strong negative inter-correlation (*r* = −0.56; Supplementary Figure 3) whereas the correlations between the remaining pairs of RR interval and HRV measures were relatively low (<0.26; Supplementary Figure 3).

### Relationship Between BOLD Fluctuations and Cardiac Dynamics

**Figure 2:**
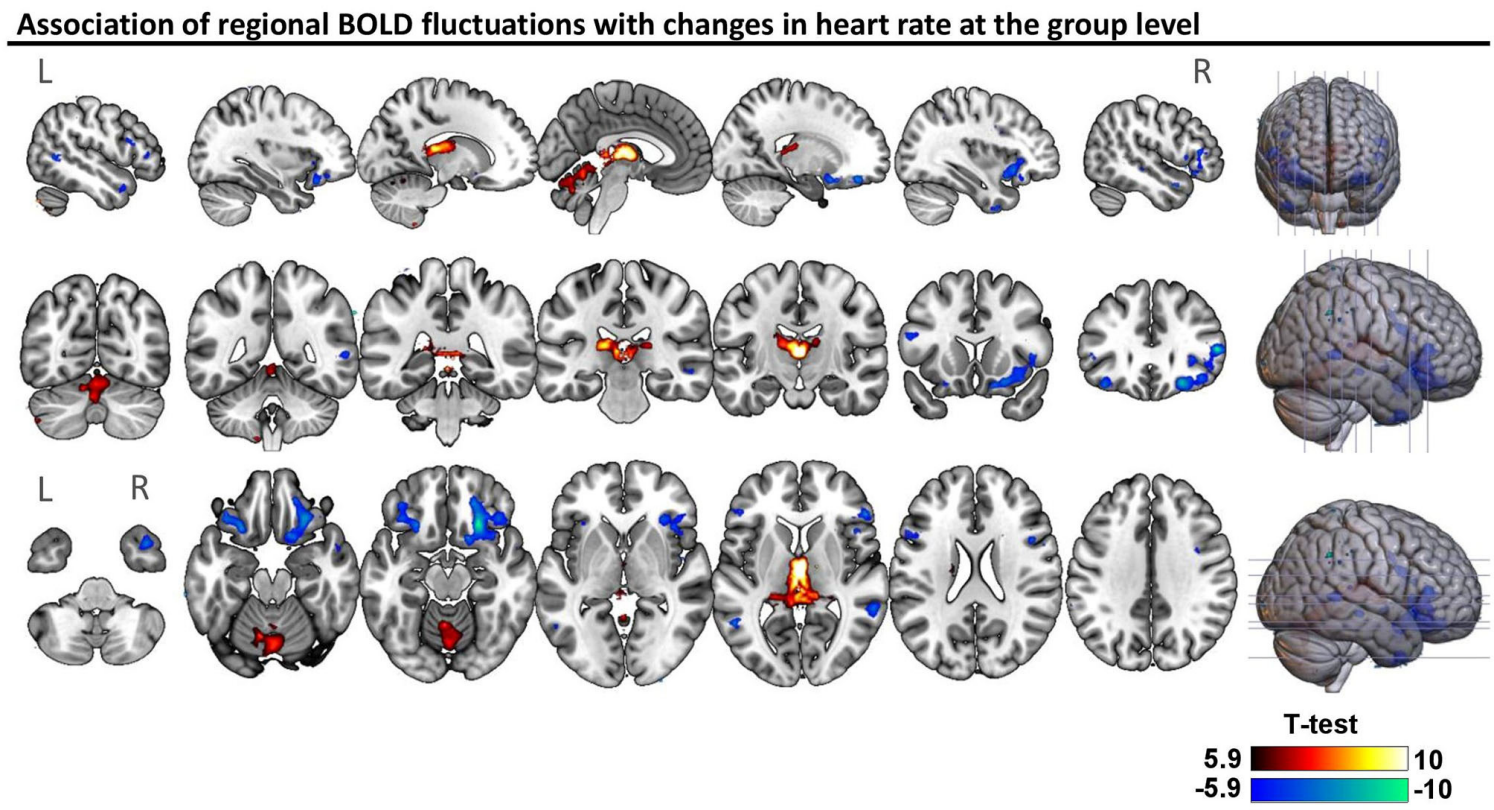
Association of regional BOLD fluctuations with changes in heart rate at the group level. Statistical map of one-sample *t*-test considering patients and controls (*n* = 44), thresholded with a voxel-wise threshold of family-wise error (FWE) rate *p* < 0.05 corrected for multiple comparisons using the random-field theory (51). The unthresholded statistical map is available at https://neurovault.org/collections/9452/.

Across all subjects, BOLD signal fluctuations were associated (*p* < 0.05; FWE-corrected) with only one of the cardiac dynamic metrics, namely instantaneous heart rate, in the following regions: positive correlations in the thalamus and several regions of the cerebellum (culmen, declive, uvula, nodulus and inferior semilunar lobule); negative correlations in the bilateral inferior frontal gyrus, orbitofrontal cortex, middle temporal gyrus, precentral gyrus and claustrum, as well as right insula and putamen (Figure 2). A more liberal threshold of *p* < 0.001 uncorrected indicated positive correlations of heart rate in bilateral caudate, and negative correlations in left insula and putamen, as well as bilateral superior temporal gyrus (Supplementary Figure 4). We did not find significant differences in correlations between the two groups.

### HRV State-Dependent Functional Connectivity

**Figure 3:**
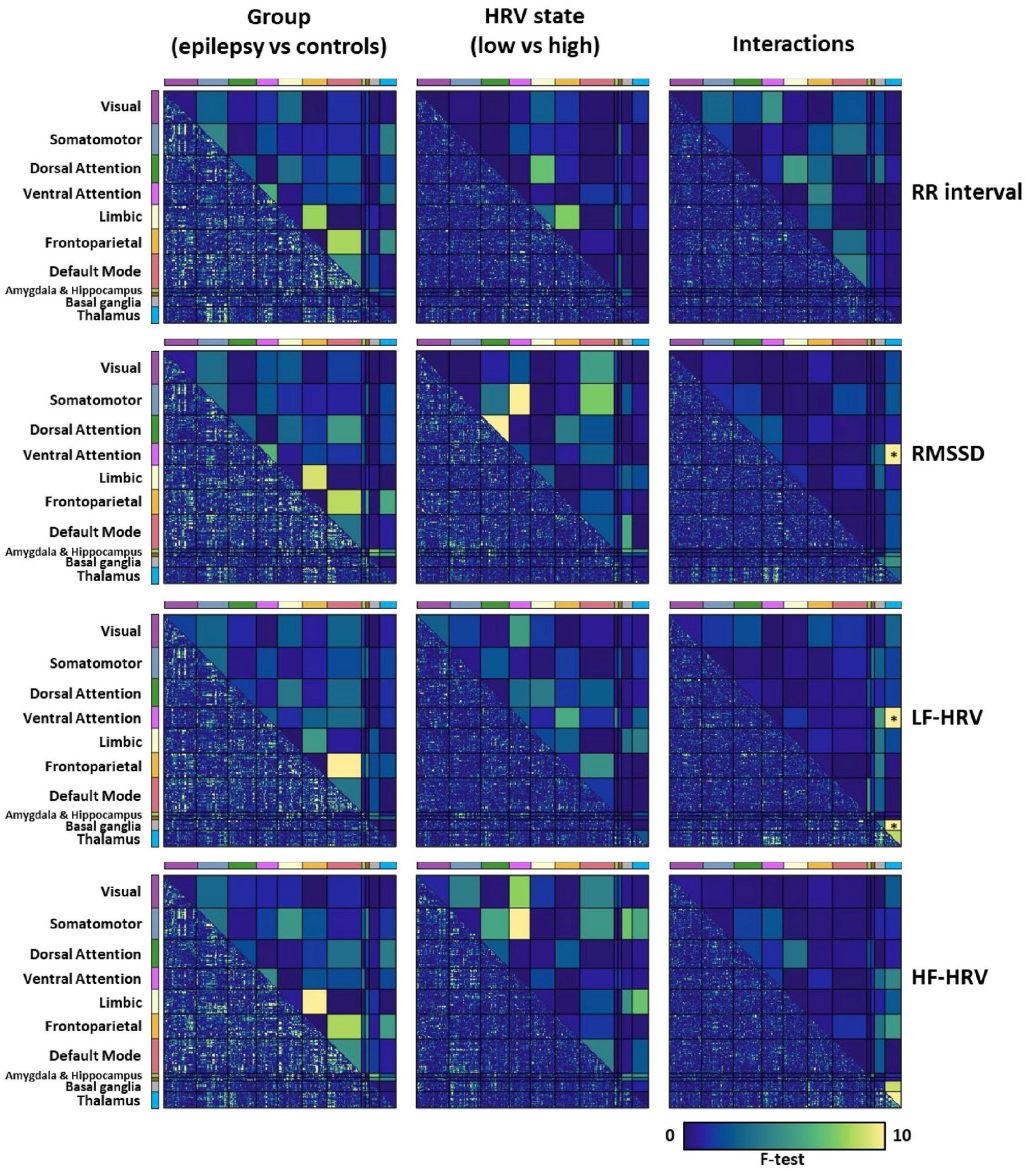
Mixed analysis of variance (ANOVA) for evaluating the effects of the group (epilepsy vs. controls; 1st column) and HRV state (low vs. high, patients and healthy controls; 2nd column) on whole-brain connectivity, and their interactions (3rd column). The lower triangular matrix corresponds to the *f*-test for pairs of parcels whereas the upper triangular matrix corresponds to the *f*-test for pairs of the eleven networks. The connectivity strength between pairs of networks that was used in the mixed ANOVA was defined as the mean correlation averaged across all pairs of parcels that belonged to the two corresponding networks. Pairs of networks with *p* < 0.0125 after FDR correction are indicated with an asterisk (*). Note that the lower triangular matrices are only shown to provide a qualitative description of the interactions of networks at the parcel level, and that their significance levels are not assessed. We observe that the connectivity strength of the thalamus with the ventral attention network and basal ganglia has strong interactions between the group and the LF-HRV state, which led us to examine thalamic connectivity with the ventral attention network and basal ganglia more carefully.

When comparing whole-brain connectivity between patients and healthy controls, for all cardiac dynamic metrics, the strongest differences were observed in the connectivity strength between the frontoparietal, limbic and default mode networks, albeit these did not reach statistical significance (Figure 3 left column). The connectivity strength of the thalamus with the ventral attention network and basal ganglia had strong interactions of group and LF-HRV state (*p* < 0.0125, FDR corrected; Figure 3 right column). The connectivity between the thalamus and ventral attention network demonstrated also strong interactions of group and RMSSD state.

**Figure 4:**
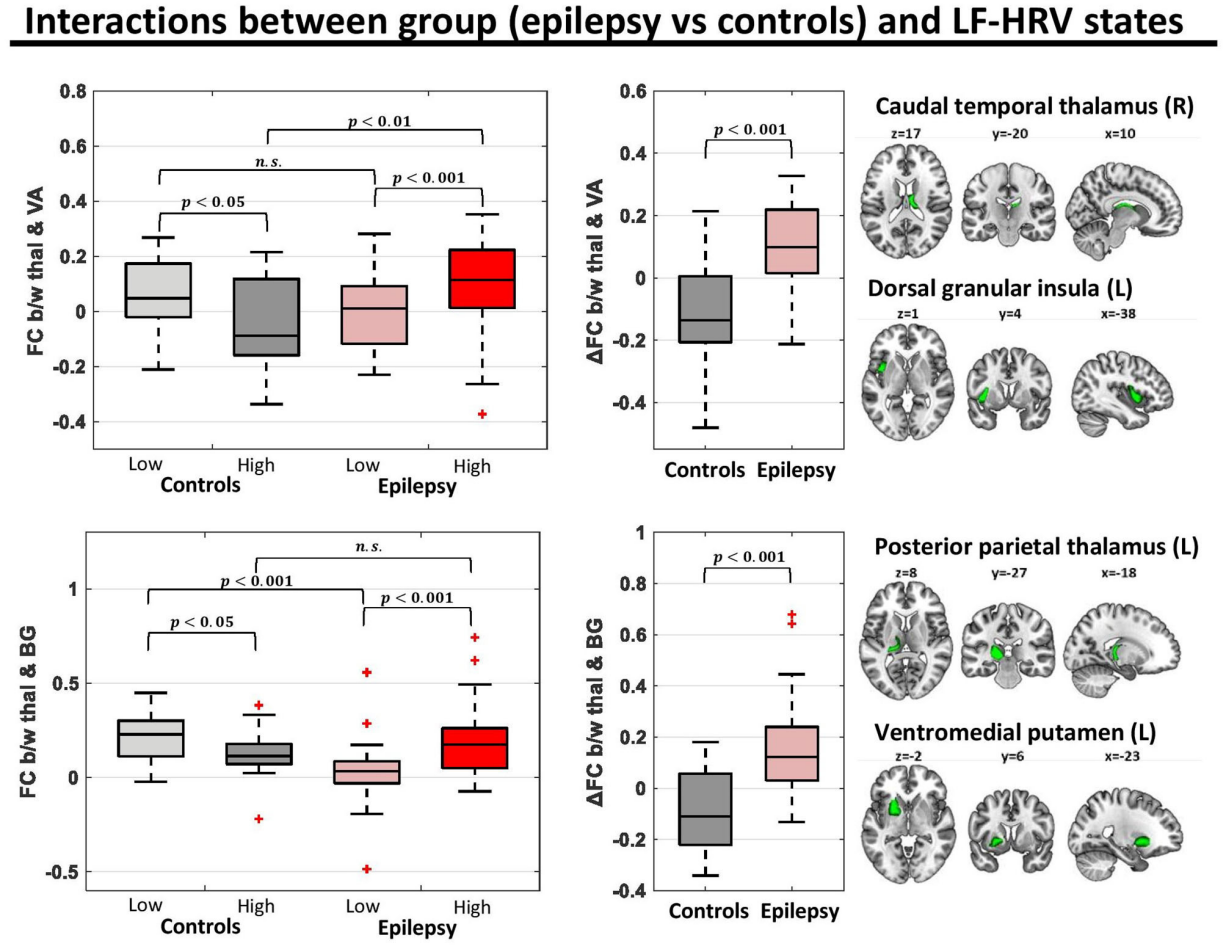
Functional connectivity (FC) for pairs of parcels with strong group (epilepsy vs. controls)-LF-HRV interactions. In the healthy controls (*n* = 16), the connectivity strength between the right caudal temporal thalamus and left dorsal granular insula [region of ventral attention (VA) network] was lower in times with high levels of LF-HRV (i.e., levels of LF-HRV in the highest quartile of a scan) compared to times with low levels of LF-HRV (i.e., levels of LF-HRV in the lowest quartile of a scan), whereas in people with epilepsy (*n* = 28) the connectivity strength was higher in times with high levels of LF-HRV. Similar results were observed for the connectivity strength between the left posterior parietal thalamus and the left ventromedial putamen [region of the basal ganglia (BG)].

To shed further light on the interactions of group and HRV state in the connectivity of thalamus with the ventral attention network and basal ganglia, we performed a *post-hoc* analysis on the connectivity between the pairs of parcels of the associated networks with the strongest interactions. Specifically, we investigated the connectivity between the right caudal temporal thalamus and left dorsal granular insula (parcel of the ventral attention network; *f*-test for interactions = 17.5) and the connectivity between the left posterior parietal thalamus and left ventromedial putamen (parcel of the basal ganglia; *f*-test for interactions = 17.7). For both connections of the thalamus, healthy controls exhibited a lower (toward negative values) connectivity strength in the high LF-HRV state compared to the low LF-HRV state, whereas epilepsy patients exhibited the opposite trend (Figure 4).

**Figure 5:**
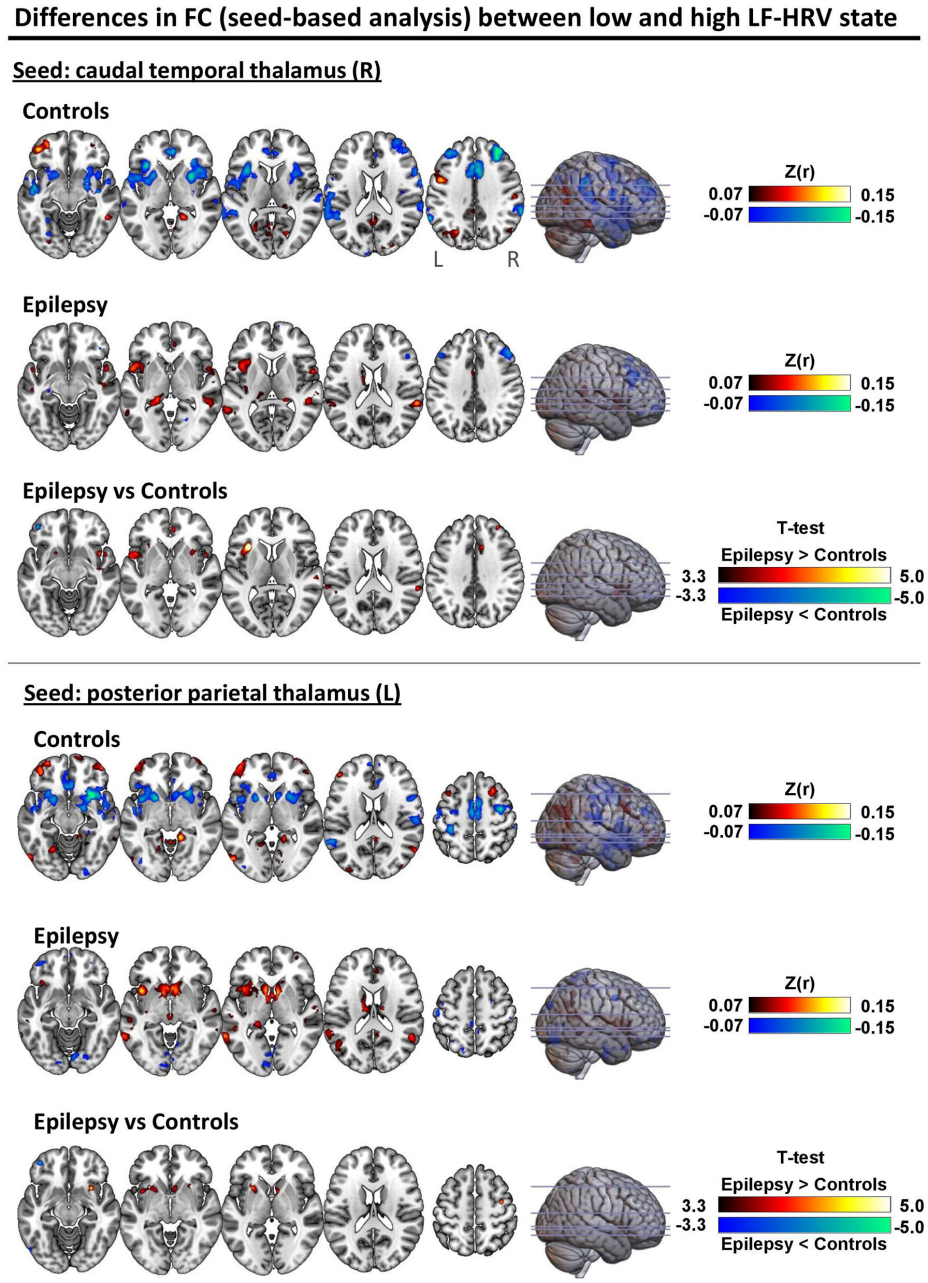
Differences in seed-based FC between low and high LF-HRV state with seeds placed in the (top) right caudal temporal thalamus and (bottom) left posterior parietal thalamus. The first and second rows of each panel show the fisher-transformed correlations average across all healthy controls and epilepsy patients, respectively. Red color indicates higher (toward positive values) connectivity with the seed region in high LF-HRV state whereas blue color indicates lower connectivity. The last row of each panel shows the two-sample *t*-test thresholded at *p* < 0.001 (uncorrected). Even though no significant differences were found between the two groups after correcting for multiple comparison (FWE; *p* < 0.05), the differences observed with *p* < 0.001 (uncorrected) are consistent with the results obtained with the analysis in the atlas space (Figures 3, 4) where the spatial autocorrelation between voxels of the same parcel are implicitly taken into account. The unthresholded statistical maps are available at https://neurovault.org/collections/9452/.

Figure 5 shows the degree to which the voxel-level connectivity profile of the right caudal temporal and left posterior parietal thalamus differs between low and high LF-HRV state (red color corresponds to higher correlations in high LF-HRV compared to low LF-HRV, and vice versa for blue color), for both controls and patients as well as the differences between the two groups. When comparing high with low HRV-state, in controls we observe a decrease in the connectivity of the caudal temporal thalamus with the bilateral anterior insula cortex (AIC), the anterior cingulate cortex (ACC), middle frontal gyrus (MFG) and supramarginal gyrus (SMG); whereas, in patients, we observe a small decrease in the connectivity with MFG and an increase with ACC. When examining the effect of LF-HRV state in the connectivity of the posterior parietal thalamus, in controls, a decrease in connectivity with the bilateral AIC, putamen and caudate appears, and in patients, an increase in connectivity with left putamen and AIC as well as bilateral caudate.

## Discussion

We examined the association of autonomic cardiac regulation with spontaneous fluctuations in fMRI and whole-brain FC in people with drug-resistant epilepsy, compared to healthy controls. In both groups, heart rate was positively correlated with fMRI signal intensity in bilateral thalamus and regions of the cerebellum, and negatively correlated with lateral regions, including bilateral inferior frontal gyrus, orbitofrontal cortex, middle temporal gyrus and right insula and putamen (Figure 2; Supplementary Figure 4). In addition, fluctuations in RMSSD and LF-HRV exhibited strong associations with changes in FC (Figure 3), despite the absence of correlation with brain activity in individual regions. Importantly, these relations differed between healthy controls and epilepsy patients. In controls, increased levels of RMSSD and LF-HRV were associated with declines in connectivity between thalamus and ventral attention network, whereas in patients, similar HRV changes accompanied increases in connectivity. The different patterns between the two groups were more pronounced for the connectivity between right caudal temporal thalamus and left dorsal granular insula (Figure 4). Note, however, that the interactions between ipsilateral regions [left (right) caudal temporal thalamus with left (right) dorsal granular insula] were also statistically significant, albeit with slightly higher *p*-values (*p* < 0.01). Therefore, it is unclear whether the stronger interactions observed between the right caudal temporal thalamus and left dorsal granular insula compared to ipsilateral interactions have some biological significance. Similar altered interactions emerged for changes in LF-HRV levels and connectivity between the thalamus and basal ganglia, with more pronounced effects for the connectivity of left posterior parietal thalamus and ventromedial putamen.

These findings support the role of thalamus, insula and putamen in autonomic control, as shown in previous studies (17, 20, 30, 53), and add roles for the temporal gyrus whose role in cardiac regulation has been recently suggested (29, 37). Despite the well-established association of activity in amygdala with heart rate fluctuations in task-based experiments (17, 54), no association was observed here. Valenza et al. (29), who also investigated the neural substrates of heart rate in task-free fMRI, found no association of amygdala activity with heart rate either, which may indicate that recruitment of amygdala activity with heart rate occurs mainly during emotional processing tasks rather than the neutral conditions studied; the amygdala traditionally serves affective roles. The neural correlates of heart rate found in our work and in Valenza et al. (29) were not entirely consistent, which may result from the more aggressive physiological correction applied in our work. Artifacts due to cardiac pulsatility were removed using the newly proposed cardiac pulsatility model (55) and systemic low-frequency oscillations were removed through gray matter signal regression (47, 56).

In healthy controls, a seed-to-voxel connectivity analysis revealed that thalamic activity was anticorrelated with core regions of the ventral attention network such as the insula, anterior cingulate cortex (ACC) and supramarginal gyrus, and this anti-correlation was enhanced during elevated levels of HRV (Figure 5). However, the HRV-dependent interplay between the thalamus and ventral attention network was absent in epilepsy. Burianová et al. (57) previously demonstrated a disturbed (static) connectivity between thalamus and the ventral attention network (also referred to as the salience network) in patients with mesial temporal lobe epilepsy which is consistent with our findings (i.e., the insula exhibited increased connectivity with the thalamus and decreased connectivity with the dorsal ACC). However, the present study also shows a strong relationship within healthy individuals between autonomic cardiac regulation and thalamus – ventral attention network connectivity, in line with findings of Chang et al. (27), which is altered in epilepsy.

The thalamus consists of a series of nuclei which are responsible, among others, for the relay of information from cardiovascular receptors to the insular cortex (31). In turn, the insular cortex integrates this information with inputs from ACC, amygdala and high-order polysensory cortex, providing interoceptive awareness. Stimulation of the insula (58, 59), basal ganglia or thalamus (60) lead to marked changes in heart rate and blood pressure. Any impairment in connectivity between these regions, such as found here, may be involved in cardiac rate and variability dysfunction.

A growing body of evidence from functional and structural studies suggests thalamic dysfunction in epilepsy which may underlie the abnormal connectivity of the thalamus with the ventral attention network and the basal ganglia observed in our study (Figures 4, 5). Allen et al. (32), using resting-state fMRI, showed that the nodal participation of thalamus, a measure that reflects the connectivity strength of a region with regions from separate large-scale networks, was increased in epilepsy patients compared to healthy controls, and particularly in patients that succumbed to SUDEP or were at high-risk. Similarly, two recent studies reported altered thalamocortical connectivity (61) as well as impaired connectivity between thalamus and basal ganglia (62) in individuals with focal to bilateral tonic-clonic seizures (FBTCS), a group of epilepsy patients associated with increased risk of seizure-related injuries and sudden unexpected death. Structural studies have revealed association of thalamic volume loss with SUDEP and high-risk patients (63) as well as with patients that present severe hypoxia during generalized tonic-clonic seizures (64). Moreover, electrical stimulations of the anterior nucleus of the thalamus has been shown in clinical trials to reduce seizure frequency even when seizures are remote from the stimulation site (65–67). The body of thalamic evidence on mediating seizure processes, and especially the altered FC between the thalamus and ventral attention network in epilepsy suggest a target for intervention. Specialized regions within the thalamus can be modified by peripheral somatosensory stimulation; activation of those thalamic sites by active stimulation has the potential to modify these FC networks, and thus alter the dysfunction patterns we found here.

RR intervals and, to a less extent, the high frequency component of HRV (i.e., HF-HRV), were on average lower in patients compared to controls (Figure 1) which is consistent with the increased interictal heart rate and decreased HRV reported in several studies (6–9). A major component of HF-HRV (0.15–0.40 Hz) is respiratory sinus arrhythmia, a phenomenon where heart rate fluctuates in synchrony with the breathing cycle at around 0.3 Hz, and is often considered to reflect parasympathetic influences on heart rate (68). Therefore, the findings may indicate reduced parasympathetic influences on the heart in the patients of our cohort. Interestingly, although RMSSD and LF-HRV had similar levels in the two groups, when state-dependent FC was assessed based on these two metrics it revealed different connectivity patterns between the groups, suggesting that HRV-state dependent FC has the potential to lead to more sensitive biomarkers for cardiac dysfunction processes than HRV quantification alone.

This study has limitations that should be considered. While cardiovascular mechanisms are likely impaired in epilepsy and contribute to SUDEP (69), breathing disturbances also appear (70), and may contribute to alterations in FC. Cardiorespiratory arrests monitored via video-electroencephalogram (VEEG) suggest that terminal cardiac arrest was preceded by central apnea in the majority of the cases (71), indicating a potential mediator role for disturbed breathing in cardiac dysfunction. To obtain a more holistic understanding of the neural processes underlying autonomic dysregulation in epilepsy, recognition of the close coupling of respiratory and autonomic control mechanisms should be incorporated in the analysis which was not possible in the present study, as breathing was not monitored during the fMRI scans. Respiratory measures would also be helpful in distinguishing parasympathetic from sympathetic activity in frequency-based HRV measures. HRV parasympathetic activity, lying within the high-frequency range (0.15–0.40 Hz) and associated with respiratory sinus arrhythmia, can decline below 0.15 Hz during periods with low breathing rate, and apnea can completely disrupt respiratory sinus arrhythmia measures. As a consequence, HRV-based measures of parasympathetic and sympathetic activity may be blurred when considering solely cardiac recordings (72).

Several studies describe an inverse relationship between heart rate and HRV measures (73–76). However, as this relationship is not well-understood and heart rate (or RR interval) is already a good measure of ANS activity that can be easily measured, further research is needed to understand the additional information provided with HRV compared to heart rate (73, 77). To this end, in this study, the power spectral density of the HRV that the LF-HRV and HF-HRV measures were derived from, was estimated using the successive difference in RR intervals rather than the RR intervals as this was found to yield HRV measures less correlated with fluctuations in RR interval. In addition, to further disentangle fluctuations in HRV from fluctuations in RR interval, the three HRV measures were orthogonalized with respect to fluctuations in RR interval.

An important caveat of this study in the use of fMRI as a means to study the neural correlates of ANS activity is that there are not well-established methods for disentangling neuronal from physiological effects of autonomic activity (18, 19). While the BOLD (T_2_*) contrast mechanism used in fMRI is a measure sensitive to changes in blood oxygenation induced by local neuronal activity (78), it is also prone to sources of noise that can be categorized to scanner artifacts, motion artifacts, high-frequency physiological artifacts and systemic low-frequency oscillations (79–81). Sources from the first three categories, including fast effects of cardiac pulsatility (~1.0 Hz) and breathing motion (~0.3 Hz), can be mitigated to a large degree using advanced pulse sequences (e.g., multi-echo fMRI) and noise correction techniques (43–45, 47, 79, 82, 83). However, systemic low-frequency (<0.1 Hz) oscillations which typically refer to BOLD fluctuations driven by changes in heart rate, breathing patterns and blood pressure can be difficult to be separated from neuronal fluctuations as they share the same mechanism; i.e., both neuronal (in an attempt to satisfy increased demands in oxygen) and physiological processes (e.g., heart rate) influence the levels of blood oxygenation (56, 84–87). When studying the neural substrates of the ANS, this is particularly problematic as brain regions not involved in autonomic regulation may share similar BOLD activity with core regions of the CAN due to effects of heart rate in global cerebral blood flow, and therefore the physiological effects of autonomic activity (e.g., fluctuations in heart rate) may lead to artificial connectivity. In this study, to mitigate the effects of systemic low-frequency oscillations, we employed gray matter signal regression which outperforms alternative preprocessing strategies (45, 47, 88, 89). Note that the effects of systemic oscillations are more prominent in visual and sensorimotor areas (85, 89), regions that did not appear to be correlated with heart rate variations in the present study (Figure 2), suggesting that the preprocessing strategy employed here successfully removed the effects of systemic oscillations. However, we cannot exclude the possibility that gray matter regression removed signal of interest as well.

HRV impairment in epilepsy is more pronounced during nocturnal periods (14, 15) and risk for SUDEP is increased during night hours (16). These observations raise the question whether alterations in FC are also enhanced by sleep or during particular phases of the HRV circadian cycles. Note that even though participants often fall asleep during resting-state fMRI and, thus, there might be segments of fMRI data corresponding to sleep in our dataset, the low sample size (*N* = 44) impedes investigations in relation to sleep effects. The potential for more exaggerated changes in FC during sleep mandates further studies on this issue.

This study represents an exploratory, data-driven approach to investigate whether large-scale networks are involved in cardiac regulation, and is hampered by potential sleep, breathing, and circadian interactions that could interfere with understanding of important brain interactions. A hypothesis-driven analysis, controlling for these interactions may elucidate more precisely the key disruptions in autonomic processes found in epilepsy.

## Conclusion

In healthy controls and people with drug-resistant epilepsy, fluctuations in heart rate covaried with brain activity in key regions of the central autonomic network and in regions associated with cardiac regulation. Functional connectivity of the thalamus with the basal ganglia, a major autonomic regulatory site, and the ventral attention network was strongly linked to levels of LF-HRV, and that relationship differed between healthy controls and epilepsy patients. These findings support a significant role for thalamic contributions to cardiovascular impairments in epilepsy which may lead to cardiac rhythm and blood pressure failings implicated in SUDEP. Because activity in regional thalamic structures can be so readily modified by somatosensory peripheral stimulation, we speculate that the findings suggest a means to interfere with the deficient functional connectivity patterns in epilepsy.

## Data Availability Statement

The datasets presented in this article are not readily available because there is not written informed consent from the patients/participants to share their data with external investigators. Requests to access the datasets should be directed to LL, louis.lemieux@ucl.ac.uk.

## Ethics Statement

The studies involving human participants were reviewed and approved by the National Research Ethics Committee (United Kingdom; 04/Q0502/89). The patients/participants provided their written informed consent to participate in this study.

## Author Contributions

MK preprocessed and analyzed data, interpreted the findings, and drafted the initial manuscript. RH interpreted the findings, revised the manuscript, and refined the clinical and physiological interpretation of findings. MG advised on methodological aspects of the study and revised the manuscript. LL and BD designed and conceptualized study, interpreted the findings, and revised the manuscript. All authors contributed to the article and approved the submitted version.

## Conflict of Interest

The authors declare that the research was conducted in the absence of any commercial or financial relationships that could be construed as a potential conflict of interest.
