# Supplementary material for: Altered Relationship Between Heart Rate Variability and fMRI-Based Functional Connectivity in People With Epilepsy
Source: Front Neurol. 2021 Jun 10;12:671890. doi: 10.3389/fneur.2021.671890 (PMC8223068; doi:10.3389/fneur.2021.671890)

Supplementary Material

Suppl. Table 1. Clinical characteristics of epilepsy patients. M=male, F=female, L=left, R=right, JME=juvenile myoclonic epilepsy, FCD=focal cortical dysplasia, MFG=middle frontal gyrus, IFG=inferior frontal gyrus, STG=superior temporal gyrus, hem=hemisphere, HS=hippocampal sclerosis, N.A.=not applicable, * subject excluded due to substantial motion.

| **Case # [sex]** | **Age at time of scan (years)** | **Epilepsy syndrome** | **Disease duration**  **(years)** | **Number of GTCS per month** | **MRI findings at time of scan** |
| --- | --- | --- | --- | --- | --- |
| E1 [M] | 20 | Focal, L frontal | 9 | 2 | Normal |
| E2 [M] | 25 | Focal, L frontal | 15 | 3 | L MFG FCD |
| E3 [M] | 33 | Generalized (JME) | 20 | 1 | Normal |
| E4 [M] | 35 | Generalized | 29 | 2 | Normal |
| E5 [F]* | 18 | Generalized (JME) | 15 | 2.5 | Normal |
| E6 [F] | 26 | Generalized (JME) | 24 | 1.5 | Normal |
| E7 [M] | 49 | Focal, L frontal | 45 | 3 | Normal |
| E8 [M] | 23 | Focal, L fronto-temporal | 11 | 1 | Normal |
| E9 [F] | 37 | Focal, L frontal | 21 | 1.5 | Normal |
| E10 [F]* | 46 | Focal, L frontal | 41 | 10 | L IFG FCD |
| E11 [F] | 19 | Focal, R parietal | 17 | 2 | L parietal FCD |
| E12 [F] | 25 | Focal, R parietal | 24 | 5 | L parietal FCD |
| E13 [M] | 22 | Focal, L temporal | 18 | 8 | L HS |
| E14 [M] | 33 | Focal, L temporal | 31 | 6.5 | L HS |
| E15 [F] | 31 | Focal, L fronto-temporal | 25 | 8 | Normal |
| E16 [F] | 26 | Focal, L fronto-temporal | 17 | 10 | Normal |
| E17 [M] | 24 | Focal, L frontal | 17 | 0 | Normal |
| E18 [M] | 24 | Focal, L frontal | 20 | 0 | L frontal FCD |
| E19 [M]* | 39 | Generalized | 35 | 0 | Normal |
| E20 [M]* | 33 | Generalized | 17 | 0 | Normal |
| E21 [F] | 30 | Generalized (JME) | 17 | 0 | Normal |
| E22 [F] | 38 | Generalized | 31 | 0 | Normal |
| E23 [M] | 27 | Focal, L Parietal | 20 | 0 | L parietal FCD |
| E24 [M] | 33 | Focal, L hem | 18 | 0 | Normal |
| E25 [F] | 24 | Focal, L frontal | 21 | 0 | Normal |
| E26 [F] | 42 | Focal, L frontal | 24 | 0 | L frontal FCD |
| E27 [F] | 28 | Focal, R parieto-occipital | 21 | 0 | R parieto-occipital FCD |
| E28 [F] | 32 | Focal, R parietal | 30 | 0 | R superior parietal FCD |
| E29 [M] | 26 | Focal, L temporal | 19 | 0 | L HS |
| E30 [M] | 28 | Focal, L temporal | 23 | 0 | L STG FCD |
| E31 [F] | 19 | Focal, L frontal | 13 | 0 | L inferior frontal infarct |
| E32 [F] | 24 | Focal, L frontal | 20 | 0 | Normal |

Suppl. Table 2. Group summaries of epilepsy patients and healthy controls. SD=standard deviation, M=male, F=female, ASD=anti-seizure drug, N.A.=not applicable.

| **Variable** | **Epilepsy patients (n=28)** | **Healthy controls (n=16)** |
| --- | --- | --- |
| Age (mean ± SD) | 28.7 ± 7.0 | 30.6 ± 5.9 |
| Sex (M:F) | 14:14 | 9:7 |
| Disease duration (years) | 21.4 ± 7.2 | N.A. |
| GTCS per month (mean ± SD) | 2.1 ± 3.1 | N.A. |
| Number of ASDs (mean ± SD) | 2.7 ± 0.9 | N.A. |
| Number polytherapy | 27 | N.A. |
| Number monotherapy | 1 | N.A. |


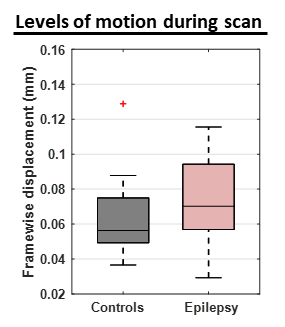


Suppl. Fig. 1. Levels of motion during the fMRI acquisition. The mean framewise displacement (FD) was on average higher in epilepsy patients (0.08 ± 0.02 mm) compared to healthy controls (0.06 ± 0.02 mm), albeit not statistically significant (*p* > 0.10). Note that four epilepsy patients were excluded due to substantial motion (mean FD > 0.25 mm) and motion-contaminated volumes (i.e. volumes with FD > 0.20 mm) were disregarded from all subjects consistent with common practices in the literature (Power et al., 2015).

Suppl. Fig. 2. FC matrices averaged across healthy controls (top row) and epilepsy patients (bottom row) considering all timepoints (excluding motion-contaminated fMRI volumes; left column), timepoints assigned to low LF-HRV state (middle column) and timepoints assigned to high LF-HRV state (right column). The average FC matrices for the low and high HRV states were very similar to the FC matrices obtained from the entire scan. Similar observations were made for the other cardiac dynamic metrics.


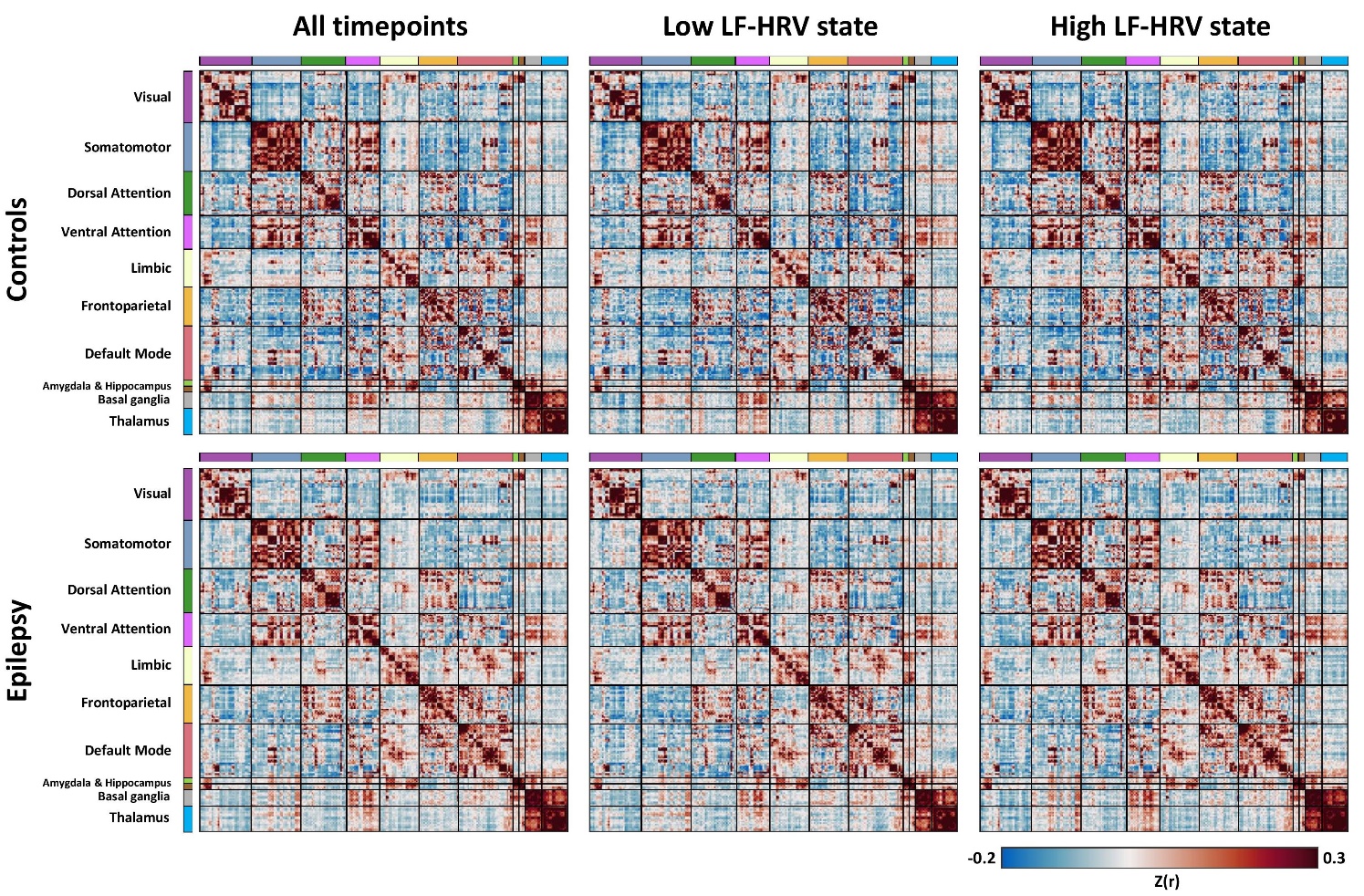

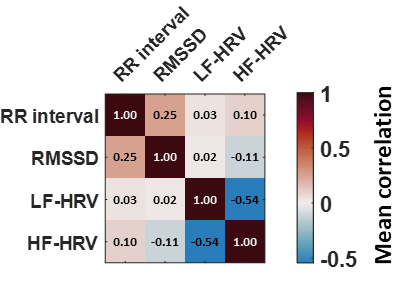


Suppl. Fig. 3. Pearson correlation coefficients between RR interval and HRV traces averaged across all subjects (n=44). Even though the time-series of HRV measures were derived from the time-series of RR intervals, they did not demonstrate strong correlations with the latter. In addition, the correlations between RMSSD and the frequency-domain HRV measures (i.e. LF-HRV and HF-HRV) were low. Despite the low correlations between RR interval and the three HRV measures, the time-series of RR interval was regressed out from the time-series of HRV measures to better characterize their unique contribution in the analysis of fMRI data.

Suppl. Fig. 4. Association of regional BOLD fluctuations with changes in heart rate at the group level thresholded at p<0.001 (uncorrected). The unthresholded statistical map is available at <https://neurovault.org/collections/9452/>.


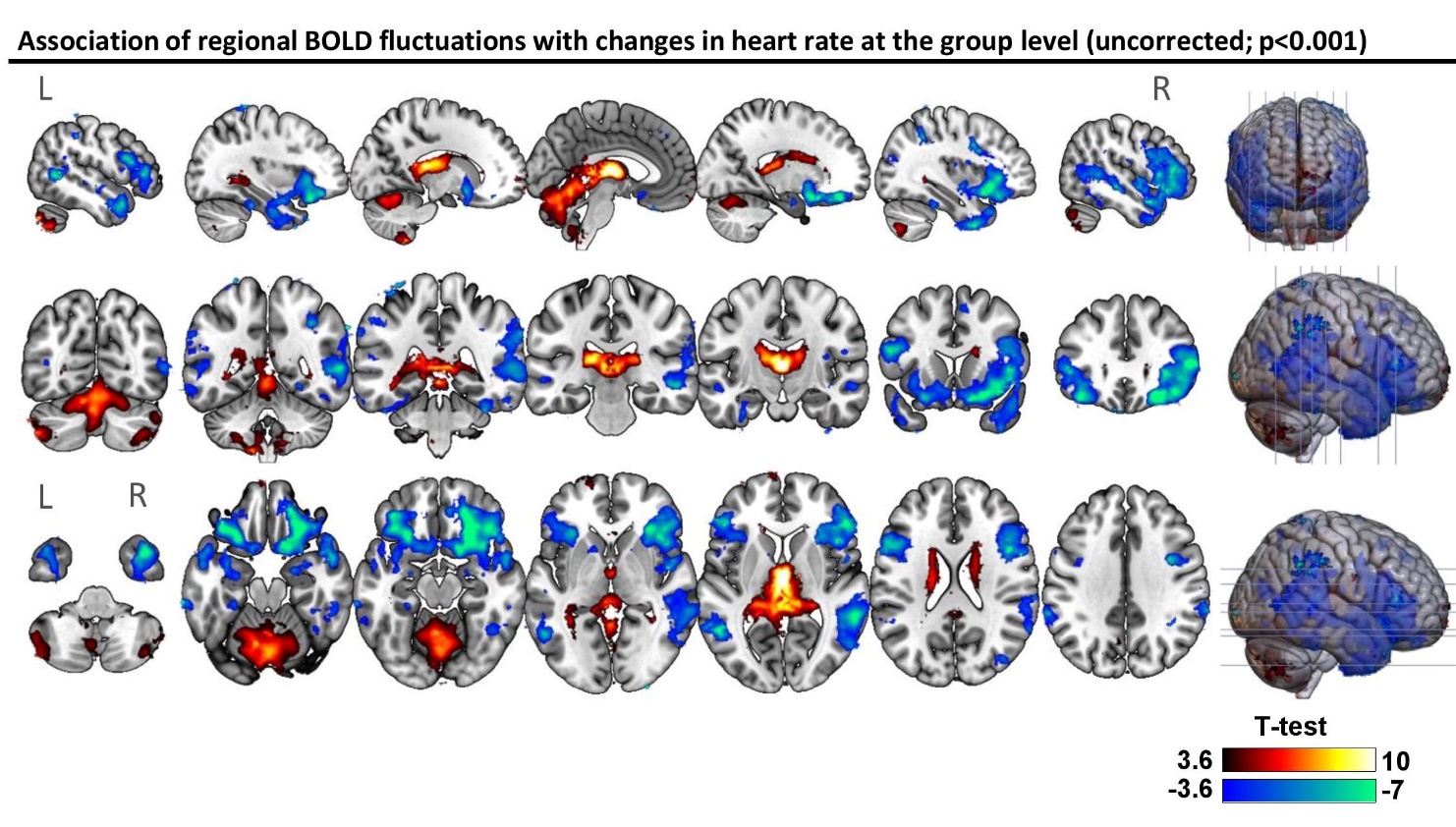

Supplement: Supplementary file 1 [file Data_Sheet_1.docx]
